# 500 ml of blood loss does not decrease non-invasive tissue oxygen saturation (StO_2_) as measured by near infrared spectroscopy - A hypothesis generating pilot study in healthy adult women

**DOI:** 10.1186/1752-2897-4-5

**Published:** 2010-05-13

**Authors:** Victor Jeger, Stephan M Jakob, Stefano Fontana, Martin Wolf, Heinz Zimmermann, Aristomenis K Exadaktylos

**Affiliations:** 1 Department of Emergency Medicine, University and University Hospital of Berne (Inselspital), Berne, Switzerland; 2 Department of Intensive Care Medicine, University and University Hospital of Berne (Inselspital), Berne, Switzerland; 3 Blood Donation Service SRK, Berne, Switzerland; 4 Biomedical Optics Research Laboratory, Clinic of Neonatology, University Hospital Zurich, Switzerland

## Abstract

**Background:**

The goal when resuscitating trauma patients is to achieve adequate tissue perfusion. One parameter of tissue perfusion is tissue oxygen saturation (StO_2_), as measured by near infrared spectroscopy. Using a commercially available device, we investigated whether clinically relevant blood loss of 500 ml in healthy volunteers can be detected by changes in StO_2 _after a standardized ischemic event.

**Methods:**

We performed occlusion of the brachial artery for 3 minutes in 20 healthy female blood donors before and after blood donation. StO_2 _and total oxygenated tissue hemoglobin (O_2_Hb) were measured continuously at the thenar eminence. 10 healthy volunteers were assessed in the same way, to examine whether repeated vascular occlusion without blood donation exhibits time dependent effects.

**Results:**

Blood donation caused a substantial decrease in systolic blood pressure, but did not affect resting StO_2 _and O_2_Hb values. No changes were measured in the blood donor group in the reaction to the vascular occlusion test, but in the control group there was an increase in the O_2_Hb rate of recovery during the reperfusion phase.

**Conclusion:**

StO_2 _measured at the thenar eminence seems to be insensitive to blood loss of 500 ml in this setting. Probably blood loss greater than this might lead to detectable changes guiding the treating physician. The exact cut off for detectable changes and the time effect on repeated vascular occlusion tests should be explored further. Until now no such data exist.

## Introduction

The goal of resuscitation is to achieve adequate tissue perfusion. The patient's history, ongoing bleeding and clinical signs of hypovolemia are the first trigger for volume substitution. Tissue perfusion is conventionally monitored using arterial base deficit and serum lactate, but these laboratory parameters are not always immediately available at the point-of-care. Additionally, relevant blood loss may not be detected by conventional hemodynamic monitoring [1].

Therefore, in the trauma setting, there is growing interest in non-invasive techniques measuring tissue perfusion. Near infrared spectroscopy (NIRS) has become a widely used method for tissue hemoglobin oxygen saturation (StO_2_) measurement in muscle and has been validated in animals [2-4] and in humans [5-11].

NIRS uses light with a wavelength of 650-950 nm and easily crosses biological tissues. Near infrared light is mainly absorbed by hemoglobin and only monitors vessels with a diameter <1 mm, because the blood levels in larger vessels are too high to reflect enough light [12]. In this setting, NIRS measurements primarily indicate the venous oxyhemoglobin concentration, as only 20% of blood volume is arterial and the NIRS device does not differentiate between systole and diastole [13].

NIRS can be used to observe the microcirculatory reactivity of peripheral tissue after a standardized ischemic event. It has been shown that StO_2 _recovery after transient occlusion of the brachial artery differs in hemodynamically unstable trauma patients in comparison to healthy volunteers [14]. The StO_2 _response after a vascular occlusion test is an example of functional hemodynamic monitoring, in which the response of the specific system to a pre-determined stress is the monitored variable. After induction of an artificial ischemic stress, local metabolic demand and reperfusion reserve can be assessed from the changes in parameters [14].

The aim of this study was to investigate the impact of a controlled and clinically relevant blood loss (500 ml) on resting StO_2 _values, as well as microcirculatory reactivity triggered by a vascular occlusion test in healthy blood donors. 500 ml of blood donation correspond to a loss of 10% of the whole blood volume. 10% of blood loss is considered to be the cut off for clinical relevant blood loss according to the current literature and may become relevant if pre- existing morbidity exists. For example, postpartum hemorrhage, defined as blood loss more than 500 ml after a vaginal delivery, is a major cause of maternal morbidity and mortality [15]. Other authors have been able to detect 500 ml of blood loss with NIRS in various settings [16,17].

We have decided to investigate the 500 ml blood loss because of the controlled and safe study setting and because higher blood volume losses are anyway related with detectable changes in physiological parameters (e.g. blood pressure, pulse rate). For the clinician, "borderline" patients without obvious signs of blood loss present the greater challenge.

We hypothesized that NIRS after a vascular occlusion test would be able to detect this amount of blood loss, because of the industries promise of "earliest" detection of volume depletion.

## Methods

### Volunteers

We investigated 20 healthy female blood donors and 10 healthy female medical students as control subjects. The study was restricted to women, as they have lower body mass index (BMI) than men and are therefore more prone to show slight symptoms of hypovolemia after blood donation.

Blood donors had a median age of 30.5 y (range 19 - 62). Their BMI was 21 kg/m^2 ^median (range 18 - 23.5). The median blood volume is estimated as 70 ml/kg body weight or 4060 ml (range 3500 - 4550). The median capillary hemoglobin was 139 g/l (range 127 - 157). This was measured once immediately after arrival at the blood donation service.

In the control group, the median age was 23 y (range 22 - 25). The median BMI was 21 kg/m^2 ^(range 19 - 23). The median blood volume was 4165 ml (range 3500 - 4480).

### Protocol

Blood donors and control subjects were in a zero degree supine position during the whole test. In both groups, there was a resting period of 5 minutes before applying the probe and performing the vascular occlusion test measurement. We measured blood pressure manually before every vascular occlusion test, using the same sphygmomanometer cuff as when occluding the artery. Blood donation started after a recovery period of 5 minutes. The second vascular occlusion test in the blood donor group started immediately after the end of blood donation.

**Figure 1:**
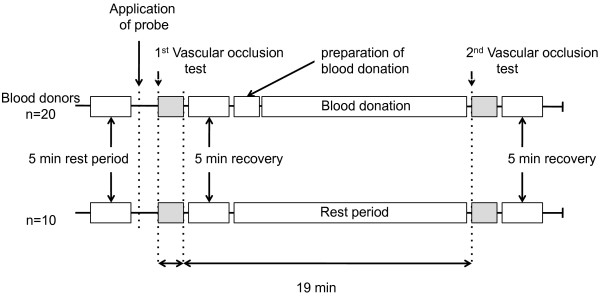
**Time axis showing course of events for both groups**.

In the control group, measurements were performed in exactly the same way as in the blood donor group. In order to detect a possible time effect from the vascular occlusion tests, a 19-minute rest period was maintained between the two vascular occlusion tests of the control group. (Fig. 1) These 19 minutes are a median (range 14 - 22 minutes) measured in the blood donor group between the end of the first and the start of the second vascular occlusion test. This period included the recovery from the first vascular occlusion, the preparation for phlebotomy and the actual blood donation, which lasted 11 minutes (range 9 - 13).

As this was an observational study aimed at controlling the quality of a freely available device, and because no additional data were collected from the volunteers, no formal ethical approval was necessary, according to our local ethical committee. Additionally, the vascular occlusion using a cuff pressure of 50 mmHg above systolic blood pressure, correlates to cuff pressures reached in non invasive (Riva Rocci) blood pressure measurement. Because no data on ethical considerations related to such kind of setting have been available, AKE and VJ have acted as volunteers. The discomfort has been within the usual limits of blood pressure measurement and was later very well tolerated by the volunteers and did not cause any discomfort.

### Measurements

We measured StO_2 _at the thenar eminence (opposite to the blood donating arm) with a commercially available tissue spectrometer (InSpectra^® ^Model 650, Hutchinson Technology, Hutchinson, Minn. US), which is promoted as being able to detect blood loss in an emergency setting [18]. The method has been previously validated [19]. The probe spacing was 15 mm.

The device measures and records StO_2 _and tissue hemoglobin index (THI) every two seconds. THI is a quantitative estimation of tissue hemoglobin concentration [20]. From these values, oxygenated hemoglobin (O_2_Hb) was obtained by multiplying StO_2 _and THI, because [19]. Accordingly, the amount of deoxygenated hemoglobin (HHb) equals the residual hemoglobin: THI - O_2_Hb = HHb. We defined baseline variables before starting a vascular occlusion test as the median over 2 minutes for StO_2_, O_2_Hb, and HHb.

**Figure 2:**
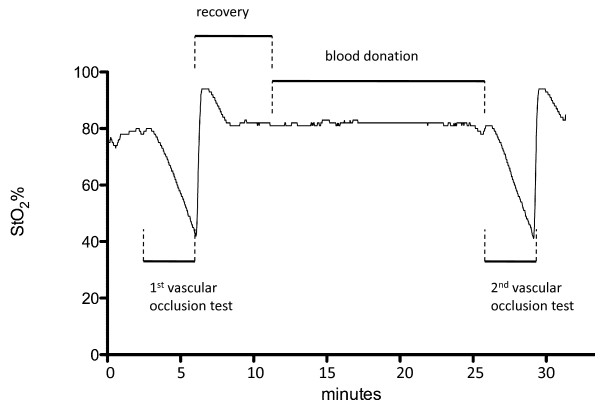
**Typical StO_2_-trace of a blood donor**. Whole length of recorded trace showing all events: 1^st ^vascular occlusion test, recovery period, blood donation and 2^nd ^vascular occlusion test. Details of a vascular occlusion test are described in figure 3.

**Figure 3:**
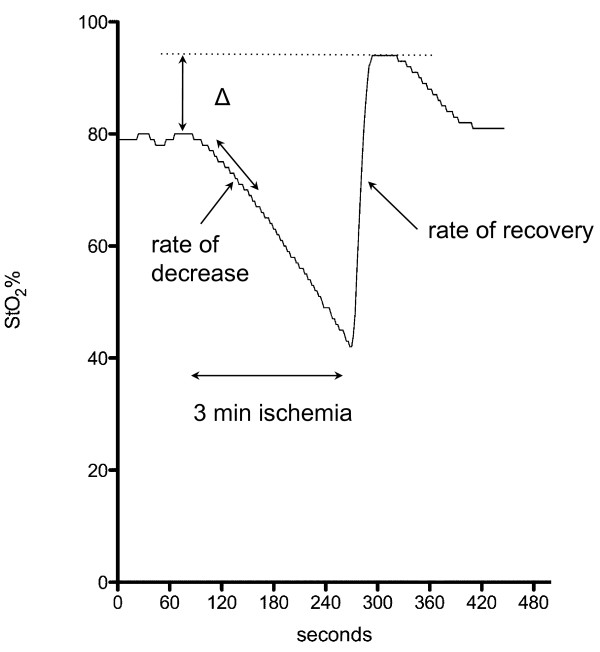
**Focus on the reaction of a StO_2_-trace after a vascular occlusion test**. Δ: delta, difference between StO_2_max and StO_2 _baseline; rate of decrease (first 25%); rate of recovery.

A vascular occlusion test was performed by inflating the sphygmomanometer cuff for 3 minutes with a pressure of 50 mmHg over systolic BP as described in detail by Creteur et al. [12]. The rate of decrease was calculated from the linear part of the decrease (first 25%) after cuff inflation [14]. The rate of recovery of StO_2 _and O_2_Hb was defined over a mean of 14 seconds (8 data points) following the nadir StO_2 _and O_2_Hb value at the end of ischemia. Delta StO_2 _and delta O_2_Hb are defined as the difference between the maximal value after reperfusion and the baseline [12]. (Fig. 2, Fig. 3)

### Data processing

Prism 5 (GraphPad Software, Inc., San Diego, CA, USA) was used to calculate the statistics. Non-parametric tests were used (Wilcoxon rank sum test for within-group differences, Mann-Whitney U-test for between-group differences). Data is presented as medians and interquartile ranges. P-values have been interpreted in an exploratory fashion.

## Results

**Figure 4:**
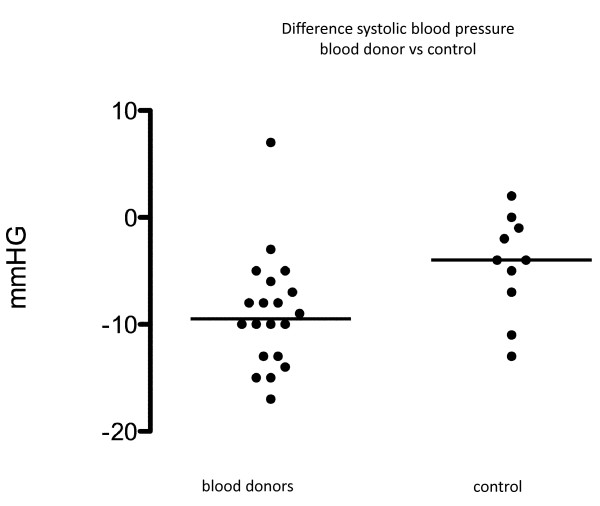
**Scatter plot showing comparison of difference in systolic blood pressure before and after blood donation (donor group) or before and after the rest period (control group)**. Line indicates median difference. p = 0.02.

There was a markedly decrease in systolic blood pressure in both groups before and after blood donation. Systolic blood pressure decreased from 118 mmHg median (interquartile range: 118 - 123) to 110 mmHg (106 - 115) (p < 0.001) in the donor group and from 112 mmHg (103 - 118) to 106 mmHg (100 - 111) (p = 0.02) in the control group. Systolic blood pressure decreased markedly more in the donor group than in the control group (p = 0.02). (Fig. 4)

For variables measured with NIRS, the differences from the first to the second vascular occlusion test did not differ between the two groups. The only exception was the differences in O_2_Hb 2 min median, which was slightly larger in the control group -0.4 (-1.0 - -0.3) than in the blood donor group -0.1 (-0.4 - 0.3) (p = 0.03). Therefore the two groups are considered to be similar and comparable.

StO_2 _and O_2_Hb 2 min median did not change substantially in the blood donor group before and after blood donation, but decreased in the control group after the rest period: StO_2 _2 min median decreased from 82.0% (79.5 - 85.0) to 80.5% (76.8 - 83.8) (p = 0.02), O_2_Hb 2 min median decreased from 10.9 (9.6 - 12.4) to 10.2 (9.4 - 11.8) (p = 0.03). HHb 2 min median did not change in the blood donor group, but increased in the control group markedly from 2.35 (2.08 - 2.56) to 2.45 (2.20 - 2.92) (p = 0.01) (Table 1).

**Table 1:** NIRS variables and systolic blood pressure before each vascular occlusion test.

Blood donors	Before blood donation		After blood donation		
	median	IQR	median	IQR	two-tailed p-value
StO_2_	79.0	74.5 - 83.5	78.5	75.0 - 83.0	0.34
O_2_Hb	10.7	9.3 - 11.9	10.8	8.9 - 11.7	0.43
deoxy Hb	2.62	2.39 - 3.24	2.70	2.34 - 4.39	0.30
SBP	118	118 - 123	110	106 - 115	<0.001*
					
**Control**	**Before rest period**		**After rest period (19 minutes)**		
	**median**	**IQR**	**median**	**IQR**	**two-tailed p-value**

StO_2_	82.0	79.5 - 85.0	80.5	76.8 - 83.8	<0.05*
O_2_Hb	10.9	9.6 - 12.4	10.2	9.4 - 11.8	<0.05*
deoxy Hb	2.35	2.08 - 2.56	2.45	2.20 - 2.92	<0.01*
SBP	112	103 - 118	106	100 - 111	<0.05*

IQR: interquartile range, *: p < 0.05

Variables measured during the vascular occlusion test did not change in the blood donor group: The rate of decrease, rate of recovery and delta for StO_2 _and O_2_Hb did not show any substantial difference before and after blood donation. In the control group, StO_2 _variables did not change markedly - in contrast to the O_2_Hb values. O_2_Hb rate of decrease was less steep after the rest period (before: -0.039 (-0.051 - -0.026); after: -0.031 (-0.046 - -0.020); p < 0.01), whereas O_2_Hb rate of recovery (before: 0.49 (0.39 - 0.63); after: 0.54 (0.39 - 0.69); p = 0.05) and O_2_Hb delta (before: 3.88 (2.43 - 4.20); after: 4.0 (3.32 - 5.72); p = 0.01) increased after the rest period (Table 2).

**Table 2:** NIRS variables during the vascular occlusion tests.

Blood donors	Before blood donation		After blood donation		
	median	IQR	median	IQR	two-tailed p-value
StO_2 _rate of decrease(%/sec)	-0.19	-0.21 - -0.16	-0.19	-0.23 - -0.15	0.78
StO_2 _rate of recovery (%/sec)	3.04	2.57 - 3.45	3.29	2.79 - 3.48	0.25
StO_2 _delta (%)	14.0	12.0 - 15.8	13.5	12.0 - 18.0	0.36
					
O_2_Hb rate of decrease (/sec)	-0.033	-0.043 - -0.026	-0.027	-0.055 - -0.021	0.98
O_2_Hb rate of recovery (/sec)	0.59	0.50 - 0.72	0.63	0.48 - 0.74	0.59
O_2_Hb delta	4.33	3.47 - 4.97	4.76	4.24 - 5.42	0.06
					
**Control**	**Before rest period**		**After rest period (19 minutes)**		
	**median**	**IQR**	**median**	**IQR**	**two-tailed p-value**

StO_2 _rate of decrease (%/sec)	-0.17	-0.19 - -0.15	-0.15	-0.20 - -0.12	0.43
StO_2 _rate of recovery (%/sec)	2.64	2.00 - 2.88	2.43	2.00 - 3.47	0.29
StO_2 _delta (%)	12.5	11.3 - 13.3	13.5	11.5 - 15.25	0.10
					
O_2_Hb rate of decrease (/sec)	-0.039	-0.051 - -0.026	-0.031	-0.046 - -0.020	<0.01*
O_2_Hb rate of recovery (/sec)	0.49	0.39 - 0.63	0.54	0.39 - 0.69	<0.05*
O_2_Hb delta	3.88	2.43 - 4.20	4.00	3.32 - 5.72	<0.01*

IQR: interquartile range, *: p < 0.05

## Discussion

The principal finding of this study is, that although systolic blood pressure decreased substantially, blood loss had no effect on superficial thenar StO_2 _before and after blood donation measured by NIRS. However other experimental NIRS devices have been successfully used to monitor blood loss after blood donation. Torella et al. showed that even 2% blood volume loss reduced peripheral hemoglobin oxygen saturation (PsO_2_, calf) significantly [16]. Soller et al. simulated hypovolemia using progressive lower body negative pressure and showed that hypovolemia corresponding to 400 - 500 ml of blood loss resulted in a decrease in forearm muscle oxygen saturation [17]. The same group could also show, in the same setting, that deep muscle oxygen saturation is more sensitive in detecting hypovolemia (experimental NIRS device) than superficial thenar StO_2 _(Hutchinson) [21]. Soller et al. discussed, that the location of the NIRS sensor as well as the tissue depth might be responsible for variations in results, although the technique used by the two devices is in principle the same. We did use exactly the same superficial thenar StO_2 _probe from Hutchinson as Soller in her study, which may explain why we did not detect any changes in NIRS parameters following blood loss.

A second finding of this study is that there might be an intrinsic effect of repeated vascular occlusion tests on O_2_Hb. This hypothesis is based on the increase in delta O_2_Hb and O_2_Hb rate of recovery and a decrease in oxygen consumption (O_2_Hb rate of decrease), which was measured in the control group but not in the blood donor group. This could be explained if there is a time effect of repeated vascular occlusion tests, designated as ischemic preconditioning [22]. This improves the ischemic tolerance of various tissues, including skeletal muscle, after one or more brief periods of ischemia, each followed by a short reperfusion phase. The underlying mechanisms are still not clear. One reason may be the reduction in the resting rate of oxygen consumption [23]. Although blood loss does not result in different StO_2 _or O_2_Hb variables after the vascular occlusion test, it seems to mask the effect of ischemic preconditioning in the blood donor group.

### Limitations

One limitation with our paper is the assumption that withdrawel of 500 ml of blood in a healthy volunteer represents a clinically relevant blood loss. Anyhow 10% of circulating blood volume can not be neglected.

In addition we did not supply data on base excess and/or lactate, the two parameters which are conventionally used to estimate tissue oxygenation. This would have helped to indicate whether a 500 ml blood loss was indeed relevant, but was not feasible in the giving setting of volunteer blood donation. Therefore, it is unclear as to whether the device failed to identify tissue ischemia completely, which is unlikely as the values decreased during vascular occlusion, or more likely, that no ischemia developed during blood donation.

## Conclusion

StO_2 _measured at the thenar eminence seems to be insensitive to blood loss of 500 ml in this setting. Probably blood loss greater than this might lead to detectable changes guiding the treating physician. The exact cut off for detectable changes and the time effect on repeated vascular occlusion tests should be explored further. Until now no such data exist. Finally, our findings suggest that StO_2 _measured by NIRS is not able to detect acute blood loss of 500 ml and therefore, it might not be useful to apply this device in the acute assessment of a bleeding trauma patient. However, the device could help to identify hemodynamic worsening during the ICU stay but at the moment not enough evidence exists to support this hypothesis.

## Competing interests

The authors declare that they have no competing interests.

## Authors' contributions

VJ, SF and AE conducted the study. All authors contributed to the design of the study, the analysis of the data and the writing of the manuscript. All authors have seen the original study data, reviewed the analysis of the data, and approved the final manuscript. VJ is the author responsible for archiving the study files.
